# Dietary Saturated Fatty Acids Modulate Pain Behaviour in Trauma-Induced Osteoarthritis in Rats

**DOI:** 10.3390/nu12020509

**Published:** 2020-02-18

**Authors:** Sunderajhan Sekar, Sunil K Panchal, Naga KR Ghattamaneni, Lindsay Brown, Ross Crawford, Yin Xiao, Indira Prasadam

**Affiliations:** 1 Institute of Health and Biomedical Innovation, Faculty of Science and Engineering, Queensland University of Technology, Brisbane, QLD 4059, Australia; s.sekar@connect.qut.edu.au; 2 Functional Foods Research Group, University of Southern Queensland, Toowoomba, QLD 4350, Australiagnkrb4u@gmail.com (N.K.G.); Lindsay.Brown@usq.edu.au (L.B.); 3 School of Science, Western Sydney University, Richmond, NSW 2753, Australia; 4 School of Health and Wellbeing, University of Southern Queensland, Toowoomba, QLD 4350, Australia; 5 The Prince Charles Hospital, Orthopedic Department, Brisbane, QLD 4032, Australia; r.crawford@qut.edu.au; 6 Australia–China Centre for Tissue Engineering and Regenerative Medicine, Queensland University of Technology, Brisbane, QLD 4059, Australia

**Keywords:** osteoarthritis, saturated fatty acids, pain, obesity, diet, cartilage

## Abstract

Osteoarthritis (OA) is a degenerative condition of joints, causing pain and swelling, and can be caused or worsened by trauma and obesity. The objectives of this study were to determine whether pain behaviour and progression of OA were increased in rats with trauma-induced OA fed dietary saturated fatty acids (SFA). Male Wistar rats were fed either a corn starch diet (C) or high-carbohydrate high-fat diet (H) with either 20% beef tallow or SFA (lauric (HLA), myristic (HMA), palmitic (HPA) or stearic (HSA) acids) for 16 weeks prior to and 8 weeks after excision of the medial meniscus of right knee joint to initiate OA when pain behaviour, glial activity, progression of knee OA, inflammatory mediators and signs of metabolic syndrome were assessed. Rats fed beef tallow, palmitic or stearic acids showed increased pain symptoms characterised by decreased hind paw/limb withdrawal thresholds and grip strengths and increased spinal astrogliosis and microgliosis compared to rats fed lauric or myristic acids. However, the severity of OA joint damage was unchanged by these dietary manipulations. We conclude that pain symptoms of trauma-induced OA in rats worsen with increased dietary beef tallow or palmitic or stearic acids, but improve with lauric or myristic acids, despite unchanged OA cartilage damage.

## 1. Introduction

Trauma is an important cause of osteoarthritis (OA), a complex multifactorial disease process leading to degeneration of joints [1]. In the 2017/2018 Australian Bureau of Statistics National Health Survey, 12% of females and 6.8% of males (2.2 million Australians) had arthritis with an economic impact of $3.5 billion annually [2]. The characteristic features of OA include degradation of the cartilage and sclerosis of subchondral bone, with increased synovial inflammation playing a major role in the degenerative bone disease [1,3,4]. Pro-inflammatory mediators may be released by the infrapatellar fat pad to stimulate proliferation of inflammatory cells in the synovial membrane, driving peripheral and central sensitisation in knee osteoarthritis [5]. The major symptom of OA is pain, which eventually leads to disability with increasing disease progression [6]. Complex mechanisms drive OA pain. These include activation of mechano-transducers and acid-sensing ion channels on sensory nerve fibres, sensitisation, transcriptional changes, and ectopic sprouting of sensory nerve fibre and central sensitisation involving both the spinal cord and brain [7]. Throughout the entire nociceptive pathway, glial cells such as astrocytes and microglia become activated after nerve injury and inflammation in the central nervous system [8]. This activation regulates chronic pain by changing glial cell morphology and releasing pro-inflammatory cytokines, which induce the proliferation of other glial cells and enhance transmission of pain [9,10].

Obesity, a major risk factor for OA [11], is characterised by chronic low-grade inflammation leading to excess fat deposition in adipose tissue, dyslipidaemia, hypertension, mitochondrial dysfunction and hyperglycaemia [12] which may then produce muscle damage and musculoskeletal complications including OA [13,14]. Our recent studies showed that synovial resident macrophages produce systemic and joint inflammation before cartilage damage in obese rats fed a high-carbohydrate high-fat diet [15]. Furthermore, we showed that saturated fatty acids (SFA) such as palmitic or stearic acids as long-chain SFA caused pronounced OA and metabolic syndrome, in contrast to the minimal effects of lauric and myristic acids as medium-chain SFA [16].

As increased consumption of long-chain SFA is a relevant risk factor for both metabolic syndrome and OA, we have examined the role of the different dietary SFA in the development of pain-related behaviour in trauma-induced OA. We hypothesised that increased dietary intake of these SFA might alter the pain symptoms in trauma-induced OA, with responses depending on chain length. This study assessed post-traumatic pain sensitisation behaviour and possible changes in joint structure following meniscectomy in rats fed diets with different medium-chain or long-chain SFA.

## 2. Materials and Methods

### 2.1. Rats and Diets

The experimental groups consisted of 36 male Wistar rats 8–9 weeks of age weighing 340 ± 2 g purchased from Animal Resource Centre, Murdoch, WA, Australia. The rats were randomly divided into six groups and were individually housed at the University of Southern Queensland Animal House in a temperature-controlled, 12 h light/dark cycle environment with free access to water and experimental diets. All animal procedures were approved by the Animal Ethics Committees of the Queensland University of Technology (Approval number 1500000117) and the University of Southern Queensland (Approval number 14REA010) under the guidelines of the National Health and Medical Research Council of Australia.

Rats were fed for 16 weeks either with a corn starch diet (C) or with a high-carbohydrate high-fat diet (H) [17]. The C diet contained 570 g of corn starch, 155 g of powdered rat food (Specialty Feeds, Glen Forest, WA, Australia), 25 g of Hubble, Mendel and Wakeman salt mixture (MP Biomedicals, Seven Hills, NSW, Australia), and 250 g of water per kilogram of diet. The H diet contained 175 g of fructose, 395 g of sweetened condensed milk, 200 g of beef tallow (all obtained from local food suppliers and supermarkets), 155 g of powdered rat food, 25 g of Hubble, Mendel and Wakeman salt mixture, and 50 g of water per kilogram of diet. In separate rat groups, beef tallow was replaced by 200 g per kilogram of diet with an SFA (lauric acid (HLA), myristic acid (HMA), palmitic acid (HPA), or stearic acid (HSA)). C rats were given drinking water without any additives while drinking water for H-based diets was supplemented with 25% fructose [17]. The carbohydrate intake in both C and H groups was approximately 68%. Measurements of body weight and food and water intakes were taken daily.

### 2.2. Surgery

After 16 weeks of the diets, rats were anaesthetised by intraperitoneal injection of Zoletil (tiletamine 15 mg/kg, zolazepam 15 mg/kg; Virbac, Peakhurst, NSW, Australia) and Xylazil (xylazine 10 mg/kg; Troy Laboratories, Smithfield, NSW, Australia) and partial medial meniscectomy of the right knee was performed [18]. The separate group of sham-operated rats was subjected to a similar surgical procedure in the right knee, except that the ligament and meniscus were not excised or manipulated. After the conclusion of the surgery, all rats received buprenorphine 0.05 mg/kg and gentamicin 5 mg/kg. Rats were allowed to recover for 1 week, during which time they were placed on a standard rat chow diet and given post-surgical medications. Once rats had recovered, they were placed on the same diet as before the surgery for a further period of 8 weeks.

### 2.3. Metabolic, Cardiovascular, and Liver Parameters

Feed efficiency and energy intakes were calculated from food and water intakes [17]. Abdominal circumference was measured using a standard measuring tape under light anaesthesia with Zoletil (tiletamine 10 mg/kg, zolazepam 10 mg/kg i.p; Virbac). An oral glucose tolerance test was performed on rats at the end of the protocol after overnight food deprivation [17]. Systolic blood pressure was measured under light sedation with Zoletil (tiletamine 10 mg/kg, zolazepam 10 mg/kg, i.p.) [17]. Dual-energy X-ray absorptiometry measurements were performed on all rats using a Norland XR-46 Bone Densitometer Machine (Norland Corp., Fort Atkinson, WI, USA) under light sedation with Zoletil (tiletamine 10 mg/kg, zolazepam 10 mg/kg; Virbac) by intraperitoneal injection at the end of 8 weeks and 16 weeks. Scans were examined using the manufacturer’s recommended software for use in laboratory animals (Small Subject Analysis Software, version 2.5.3/1.3.1; Norland Corp.). Body mass index was calculated as g/cm^2^.

Rats were euthanised after 25 weeks using Lethabarb^®^ (100 mg/kg pentobarbitone sodium, i.p.; Virbac). After euthanasia, blood was collected to separate plasma and the plasma was stored at −20 °C for further analysis. Hearts were isolated to perform Langendorff heart preparation to measure diastolic stiffness [17]. Following this, tissues such as liver, left ventricle (with septum), right ventricle and abdominal fat (including retroperitoneal, epididymal and omental) were removed for weighing and expressed as mg/mm of tibial length. Plasma concentrations of total cholesterol, triglycerides and non-esterified fatty acids (NEFA), and plasma activities of alanine transaminase (ALT) and aspartate transaminase (AST) were measured [17].

### 2.4. Pain Behaviour

#### 2.4.1. von Frey Test

The rats were placed in cages with a metallic mesh base, 20 cm above the bench. A habituation period of 15 min was allowed before the test. The von Frey filaments (BIO-VF-M, Bioseb, Vitrolles, France) were placed on the paw and the withdrawal threshold was determined by applying force ranging from 2–20 g. The paw sensitivity threshold was defined as the minimum gentle touch required to elicit a robust and immediate withdrawal of the paw. Positive responses included a sudden withdrawal of hind paw or flinching behaviour in response to the removal of the filaments. Voluntary movements which were associated with locomotion were not taken as a positive withdrawal response. The von Frey filaments were applied on each anterior paw with an interval of 5 s. The measurement was taken 3 times and the final value was obtained by averaging the 3 readings. A threshold of 6 g was chosen as an indication of allodynia.

#### 2.4.2. Hind Limb Grip Strength

Hind limb grip strength was measured with a rat grip strength meter (BIO-GS3, Bioseb, Vitrolles, France). Each rat was gently restrained by grasping the area around its rib cage and then was allowed to hold on to the wire mesh frame attached to the grip meter. The operator then moved the animal in a rostral-to-caudal direction until the rat lost the grip of the mesh frame. Each rat was measured 3 times at approximately 2 min interval to obtain the mean grip force (g).

#### 2.4.3. Pressure Application Measurement (PAM)

A PAM device (BIO SMALGO, Bioseb, Vitrolles, France) was used to measure the mechanical pain threshold of the knee joint. The instrument consisted of a calibrated force sensor that is worn on the operator’s thumb. The rate of application of the force is decided by the operator and the force (g) required to elicit the response (withdrawal of the limb) was recorded as a positive reading. Three consecutive measurements were taken at intervals of 5 s.

### 2.5. Assessment of Articular Cartilage, Subchondral Bone, Lumbar Spinal Cords, and Dorsal Root Ganglia

After euthanasia, the knee joints were harvested. Micro-CT was performed to analyse the subchondral bone changes. Tibia were scanned using a micro-CT (Scanco μCT 40, Scanco Medical, Switzerland) and then processed for histology [16]. Mankin scoring was performed to assess the severity of the cartilage damage [16]. The mean Mankin score from 5–6 samples from each animal was used to determine the mean ± standard deviation for each group. Two independent observers assessed cartilage damage in a blinded manner.

After euthanasia, lumbar spinal cords (SC) and dorsal root ganglia (DRG) were removed (*n* = 4 per group) and rinsed in phosphate-buffered saline and later post-fixed in 4% paraformaldehyde for 3 h. The fixed SC and DRG tissues were then embedded in paraffin and sectioned into 6 μm sections for immunofluorescence analysis. The lumbar SC and DRG (*n* = 4 per group) were rinsed in phosphate-buffered saline and stored in RNA Later solution (Ambion 7024) for real-time PCR analysis.

The SC and DRG sections underwent immunofluorescence analysis of glial fibrillary acidic protein (GFAP) [18] and ionised calcium binding adaptor molecule-1 (Iba-1) [19], which label the activated astrocytes and microglia, respectively. Briefly, the sections were deparaffinised in xylene and rehydrated in decreasing concentrations of ethanol. The tissue slices were first permeabilised with proteinase K for 30 min at 37 °C. The sections were then incubated with rabbit anti-GFAP (1:500, Abcam- ab7260) for 1 h at room temperature or with goat anti-Iba-1 (1; 500, Abcam-ab5076) for 1 h at room temperature. After primary antibody incubation, the sections were incubated with the appropriate secondary antibody (1:300, Goat anti-rabbit Alexa 488 or 1:300, Donkey anti-goat TR 566) for 1 h at room temperature. The sections were then cover-slipped using ProLong Gold Antifade Mountant with DAPI (4′,6-diamidino-2-phenylindole, P36935, ThermoFisher, Australia). For controls, the same procedures were carried out either without primary antibody or with isotype-matched IgG instead of primary antibody.

### 2.6. RNA Extraction and Real-time PCR

Total RNA was extracted using TRIzol reagent (15596018, Invitrogen). Real-time quantitative PCR [20] using SYBR Green detection was performed on the ABI 7500 Fast Real-Time PCR system (Applied Biosystems, Foster City, CA, USA). The primers used were GFAP (NCBI Reference Sequence ID: NM_017009, Gene ID: 24387) and AIF1 (NCBI Reference Sequence ID: NM_017196, Gene ID: 29427). All samples were measured in triplicate and the mean value of all experimental samples was calculated for comparative analysis. Quantitative measurements of all primers used in this study were determined using the (2^−ΔCt^) method, and 18S and β-actin expression were used as the internal controls [18,20,21].

### 2.7. Quantification of Inflammatory Markers

Blood plasma was stored frozen for later measurements of inflammatory markers using enzyme-linked immunosorbent assay (ELISA; R&D Systems, Australia). Plasma samples were thawed on ice and processed as per manufacturer’s protocol. Concentrations of IL-1β, IFN-γ and IL-10 were quantified by ELISA (rat IL-1β, IFN-γ and IL-10 Quantikine; R&D Systems, In Vitro Technologies Pty Ltd., Noble Park, VIC, Australia).

### 2.8. Statistical Analysis

Statistical analyses were performed using GraphPad Prism 7.0. The data are presented as the mean ± standard error of the mean (SEM) for metabolic data and standard deviation (SD) for bone data and were analysed with ANOVA method. Repeated-measures analysis of variance with post hoc tests (Dunnett’s/Bonferroni) was used to assess statistical significance. The level of significance was set at *p* < 0.05.

## 3. Results

### 3.1. Metabolic, Cardiovascular and Liver Parameters

**Figure 1:**
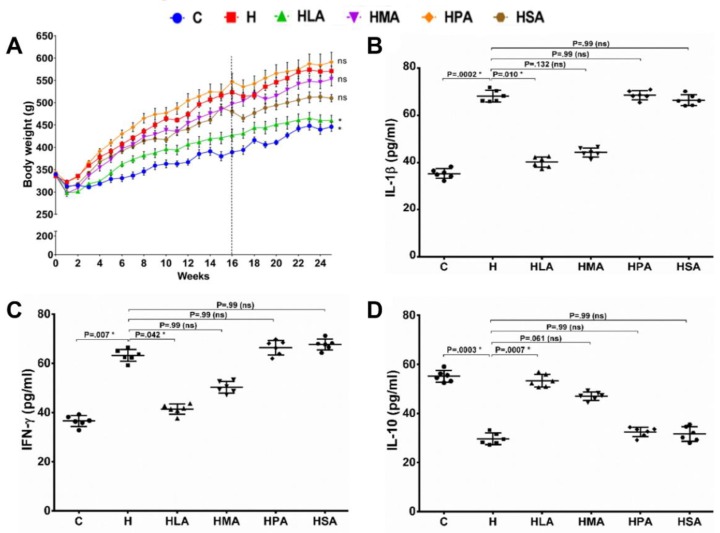
Weight gain and inflammatory markers in rat plasma. (**A**) Body weight of rats fed with the different saturated fatty acid diets for a period of 25 weeks (Dotted line showing the week of surgery); *n* = 6. * indicate *p* < 0.05 H vs. C, HLA, HMA, HPA and HPA. Plasma pro-inflammatory cytokine IL-1β (**B**), IFN-γ (**C**) and anti-inflammatory cytokine IL-10 (**D**) concentrations in different saturated fatty acids diet-fed rats. All values are represented as mean ± SD, *n* = 6; *p* < 0.05. C, corn starch diet-fed rats; H, high-carbohydrate high-fat diet-fed rats; HLA, high-carbohydrate high-lauric acid diet-fed rats; HMA, high-carbohydrate high-myristic acid diet-fed rats; HPA, high-carbohydrate high-palmitic acid diet-fed rats; HSA, high-carbohydrate high-stearic acid diet-fed rats.

At 25 weeks, the body weights of HPA, HMA and HSA rats were not different to that of H diet rats but were greater than C rats. In contrast, HLA rats showed a markedly smaller increase in body weight than H, HMA, HLA and HSA rats (Figure 1A). Plasma IL-1β and IFN-γ concentrations were higher and similar in H, HPA and HSA rats than that in C rats. Both HLA and HMA rats had lower concentrations of both inflammatory markers in comparison to H rats (Figure 1B,C). In contrast, concentrations of the anti-inflammatory cytokine, IL-10, were higher in C, HLA and HMA rats than in H, HPA and HSA rats (Figure 1D).

Food intake was higher in C rats compared to all other groups while HLA rats had the lowest food intake. Water intake was higher in HLA, HPA and HSA rats compared to the other three groups (Table 1). Energy intake was in the order of HPA = HSA > HMA = HLA = H > C. Feed efficiency was higher in H rats compared to HMA and HPA rats which was higher than in C, HLA and HSA rats (Table 1). H, HMA and HPA rats had higher abdominal circumference compared to C and HLA rats, whereas HSA rats had intermediate abdominal circumference (Table 2). Total abdominal fat was higher in H, HMA and HPA rats compared to C, HLA and HSA rats (Table 2). Bone mineral density was higher in H rats compared to HSA rats, while other groups had intermediate bone mineral density. Bone mineral content was higher in HPA rats compared to HMA rats, and H rats had intermediate bone mineral content with HPA and HMA rats. C, HLA and HSA rats had lower bone mineral content compared to other groups (Table 2).

Total body fat mass was higher in H, HMA and HPA rats compared to the other 3 groups. Lean mass was unchanged among the groups (Table 2). Basal blood glucose concentrations were unchanged between the groups due to higher variability within the groups. However, H rats showed higher area under the curve suggesting impaired glucose tolerance (Table 2). H, HMA, HPA and HSA rats had higher plasma concentrations of triglycerides and NEFA, whereas plasma total cholesterol concentrations were unchanged among the groups (Table 2). Systolic blood pressures were higher in H, HMA, HPA and HSA rats than in C and HLA rats (Table 3). Left ventricular diastolic stiffness was lower in C and HLA rats compared to other groups while left ventricular weight was higher in H rats compared to C and HLA rats, and it was intermediate in HMA, HPA and HSA rats (Table 3). Right ventricular weights were unchanged among the groups (Table 3). Liver weights were higher in H, HMA and HPA rats compared to the three other groups, whereas plasma activities of ALT and AST were unchanged among the groups (Table 3).

### 3.2. Pain Behaviour Post-Meniscectomy

The contralateral paws of rats in all groups were not different from each other at all time points and for all behavioural tests; furthermore, sham rats did not differ from control rats.

**Figure 2:**
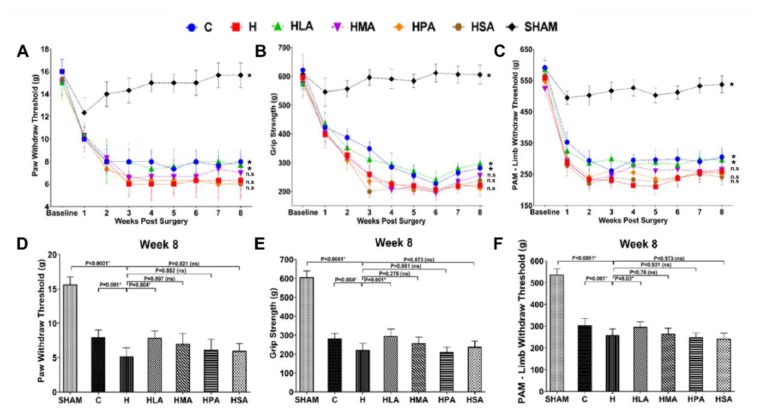
Evaluation of pain behaviour. Changes in the hind paw withdrawal threshold (**A**), grip strength (**B**) and limb withdrawal threshold (**C**) in rats fed with different saturated fatty acid diets for a period of 8 weeks post-surgery. Week 8 changes in hind paw withdrawal threshold (**D**), grip strength (**E**), and limb withdrawal threshold (**F**) in rats fed with the different saturated fatty acid diets. All values are represented as mean ± SD, *n* = 6. C, corn starch diet-fed rats; H, high-carbohydrate high-fat diet-fed rats; HLA, high-carbohydrate high-lauric acid diet-fed rats; HMA, high-carbohydrate high-myristic acid diet-fed rats; HPA, high-carbohydrate high-palmitic acid diet-fed rats; HSA, high-carbohydrate high-stearic acid diet-fed rats.

Medial meniscectomy to the ipsilateral knee induced mechanical hypersensitivity in comparison to the sham-operated knee. Rats with medial meniscectomy developed tactile allodynia measured by von Frey tests at week 1 after surgery. From week 2 to week 8, the paw withdrawal threshold (PWT) of C rats was higher than that of the H rats, while the PWT of the HPA and HSA rats did not change from week 3 to week 8, and also exhibited similar allodynia in comparison to the H rats, suggesting increased pain perception in these three groups. The HMA rats had PWT readings similar to that of the H rats. In contrast, the HLA rats showed increased PWT in comparison to H rats (Figure 2A). Similar changes were observed in terminal week (week 8) measurements, whereas in HLA rats showed increased PWT compared to other SFA diet-fed rats (Figure 2D).

Consistent with von Frey results, rats showed signs of decreased grip strength 1 week after meniscectomy. From week 2 to 8, the grip strength of C rats was higher than that of H rats. In comparison with H rats, HMA, HPA and HSA rats exhibited similar decreased grip strength and hence were not different. In contrast, the HLA rats increased grip strength compared to H rats (Figure 2B). Similar results were observed in terminal week (week 8) measurements, whereas HLA rats showed increased grip strength compared to other SFA diet-fed rats (Figure 2E).

The results of the PAM which measures mechanical limb withdrawal threshold (LWT) were consistent with von Frey and grip strength tests. C rats increased LWT in comparison with H rats. HMA, HPA and HSA rats showed a gradual decrease in LWT from week 3 through to week 6 and were quite similar to that of H rats, and hence were not different to each other. In contrast, HLA rats showed an increase in LWT throughout the 8 weeks of OA progression compared to H rats (Figure 2C). Similar results were observed in terminal week (week 8) measurements, where in HLA rats showed increased LWT compared to other SFA diet-fed rats (Figure 2F).

### 3.3. Structural Changes in Knee Joints

**Figure 3:**
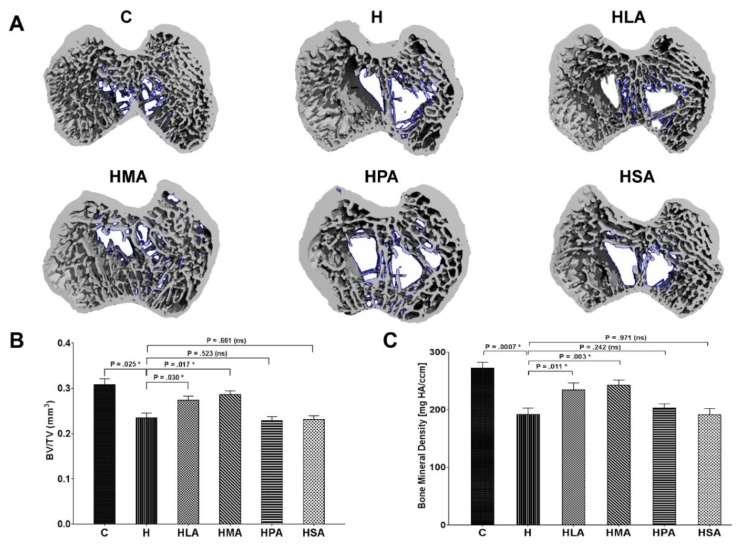
Bone volumetric analysis. (**A**) Reconstructed axial micro-CT cross-section images of the medial and lateral tibial plateau (ROI) of the knees of rats fed the different diets. For morphometric analysis, (**B**) the bone volume fraction (BV/TV) was calculated as the ratio of segmented bone volume (BV) to the total volume (TV) of the ROI. In addition, (**C**) the bone mineral density (BMD) of the ROI was also calculated. All values are represented as mean ± SD, *n* = 6. ROI, Region of interest. C, corn starch diet-fed rats; H, high-carbohydrate high-fat diet-fed rats; HLA, high-carbohydrate high-lauric acid diet-fed rats; HMA, high-carbohydrate high-myristic acid diet-fed rats; HPA, high-carbohydrate high-palmitic acid diet-fed rats; HSA, high-carbohydrate high-stearic acid diet-fed rats.

3-Dimensional micro-CT scanning showed structural changes in the subchondral bone of OA rats in the tibial plateau (Figure 3A). Examination of the subchondral bone showed that H rats had reduced BV/TV compared to that of the C group rats. HLA and HMA rats exhibited increased BV/TV compared to H rats, whereas the HPA and HSA rats showed no changes compared to H rats (Figure 3B). Consistent with BV/TV results, BMD decreased in H rats in comparison with C rats. HPA and HSA rats showed BMD similar to H rats, while HLA and HMA rats showed increased BMD compared to H rats (Figure 3C).

**Figure 4:**
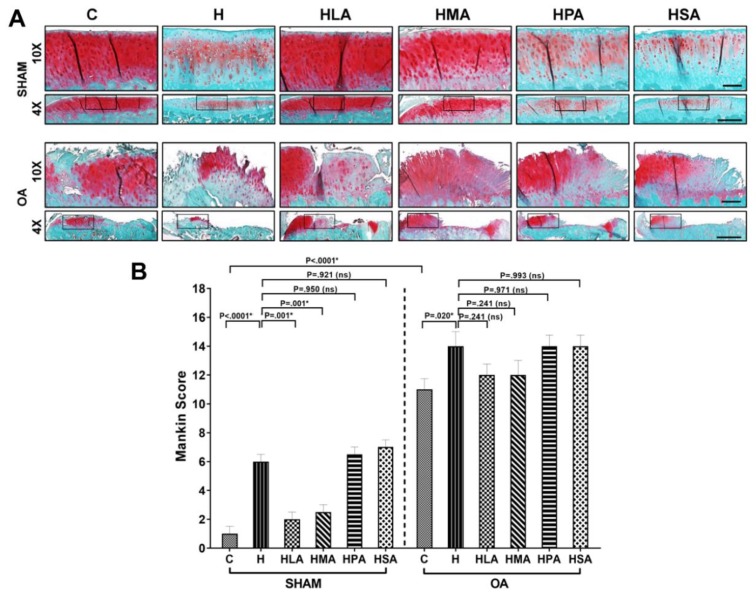
Histological evaluation of the knee joints. Pictures were obtained under 4× and 20× magnifications representing the medial tibial plateau. (**A**) Sections of articular cartilage stained with Safranin O/Fast Green shows the extent of proteoglycan loss among the different diet groups in both sham and osteoarthritic (OA) rats. (**B**) The severity of articular cartilage degradation was graded using Mankin scoring system in both sham and OA rats. All values are represented as mean ± SD, *n* = 6. C, corn starch diet-fed rats; H, high-carbohydrate high-fat diet-fed rats; HLA, high-carbohydrate high-lauric acid diet-fed rats; HMA, high-carbohydrate high-myristic acid diet-fed rats; HPA, high-carbohydrate high-palmitic acid diet-fed rats; HSA, high-carbohydrate high-stearic acid diet-fed rats.

Knees from post-meniscectomy rats exhibited OA-phenotypical cartilage changes, indicated by increased degeneration of the cartilage layer, loss of proteoglycans and fibrillation, whereas the sham-operated knees showed a normal appearance of the cartilage without any degeneration or subchondral bone changes (Figure 4A). In the sham group, the joints from the C rats showed smooth articular cartilage surface, normal cellularity and strong intensity of safranin-O staining. In contrast, sham-operated H, HPA and HSA rats showed degenerative changes of the articular cartilage especially in the surface zone, indicated by surface irregularity, loss of proteoglycans evidenced by weak safranin-O staining and increased Mankin scores. In contrast, HMA and HLA sham-operated rats showed cartilage structures, proteoglycan intensity and Mankin scores that were similar to C rats (Figure 4A,B).

In post-meniscectomy rats, the joints were not visibly different, as all groups exhibited similar damage (Figure 4A). Post-meniscectomy rats demonstrated erosion of the hyaline cartilage with increased loss of proteoglycans from the extracellular matrix, as evident by the marked reduction of safranin-O staining and increased Mankin scores compared to sham rats, independent of SFA treatment (Figure 4A,B). The articular surface of post-meniscectomy rats of H, HLA, HMA, HPA and HSA groups showed similar fissures, fibrillation and exposure of the subchondral bone, as well as the formation of cysts and presence of osteophytes.

### 3.4. Spinal Glial Cell Activation Post-Meniscectomy

**Figure 5:**
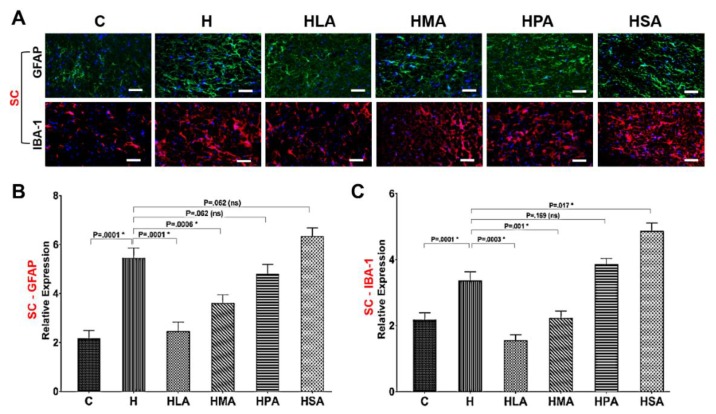
Glial cell activation in the spinal cord (SC). (**A**) Typical dorsal sections of the SC showing astrocyte (GFAP—astrocyte marker) and microglia (IBA-1—microglia marker) activation in OA rats fed the different diets. Quantification of GFAP (**B**) and IBA-1 (**C**) mRNA levels in the SC was performed using qPCR. All values are represented as mean ± SD, *n* = 4. C, corn starch diet-fed rats; H, high-carbohydrate high-fat diet-fed rats; HLA, high-carbohydrate high-lauric acid diet-fed rats; HMA, high-carbohydrate high-myristic acid diet-fed rats; HPA, high-carbohydrate high-palmitic acid diet-fed rats; HSA, high-carbohydrate high-stearic acid diet-fed rats.

**Figure 6:**
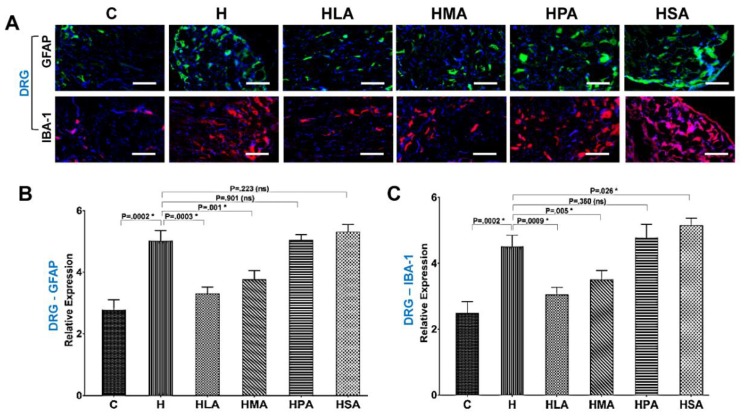
Glial cell activation in dorsal root ganglia (DRG). (**A**) Typical dorsal sections of the DRG showing astrocyte (GFAP—astrocyte marker) and microglia (IBA-1—Microglia marker) activation in OA rats fed the different diets. Quantification of GFAP (**B**) and IBA-1 (**C**) mRNA levels in the DRG was performed using qPCR. All values are represented as mean ± SD, *n* = 4. C, corn starch diet-fed rats; H, high-carbohydrate high-fat diet-fed rats; HLA, high-carbohydrate high-lauric acid diet-fed rats; HMA, high-carbohydrate high-myristic acid diet-fed rats; HPA, high-carbohydrate high-palmitic acid diet-fed rats; HSA, high-carbohydrate high-stearic acid diet-fed rats.

In addition to pain behaviour, activation of spinal astrocyte and microglia was examined in post-meniscectomy rats for evidence of astrogliosis and microgliosis in both SC and DRG. HPA and HSA rats increased activation of astrocytes (GFAP) and microglia (IBA-1) in the SC and DRG and were similar to the activation patterns of H rats. In contrast, HLA and HMA treatment reduced activation of astrocytes and microglia in SC and DRG compared to H rats (Figure 5A and Figure 6A).

The pain-associated proteins GFAP and IBA-1 which are upregulated during OA-induced knee joint pain were assessed to determine whether activation of neuronal microglia and astrocytes was linked with corresponding changes in the messenger RNA (mRNA) levels within the cellular components of SC and DRG. Real-time PCR showed that the expression of both GFAP and IBA-1 in SC and DRG was increased in H rats compared to C rats. The expression in H, HPA and HSA rats was similar but in contrast, HLA and HMA rats had decreased expression in comparison to H rats (Figure 5B,C and Figure 6B,C).

## 4. Discussion

OA is both an age- and mechanical- stress-related degenerative joint disease as well as a metabolic disease with independent effects of diabetes, dyslipidaemia and hypertension to cartilage degradation [3,22]. Animal models of OA may provide evidence for mechanisms of OA and possible interventions but there is no single, widely-accepted animal model [23]. Surgical models in rats produce joint instability, altered joint mechanics and local inflammation; these models are reproducible with rapid onset and progression which may not fully mimic human OA [23]. Non-invasive monitoring with magnetic resonance imaging of the femorotibial joint in rats following partial medial meniscectomy showed progressive changes in cartilage related to trauma-induced OA [24]. More commonly, OA is induced in rats by injection of monosodium iodoacetate into the knee or ankle joint [25]; however, this intervention produces chondrocyte death rather than trauma-induced cartilage degeneration in OA. Pain is a major characteristic of human knee OA, so measurement of pain in rat models of OA is essential for translation to humans. The advantages and disadvantages of the methods used in this project (von Frey, hind limb grip strength and mechanical pain threshold of the knee joint) have been reviewed [26,27,28]; none are perfect matches to human OA pain, but they are widely accepted as appropriate methods for use in rodents, for example in the testing of analgesics [29].

Chronic low-grade inflammation in obesity may worsen the damage and pain in OA [13,14]. Obesity-induced osteoarthritis in the knee can be modelled by surgery on rats with diet-induced obesity [30]. Abdominal obesity is part of metabolic syndrome, together with hypertension, hyperglycaemia, fatty liver, and inflammation, which increases the risk of cardiovascular disease and diabetes; this syndrome is mimicked by a diet high in simple sugars, and saturated and trans fats in rats [15]. The physiological changes in this validated dietary model of human metabolic syndrome in rats can be reversed by dietary interventions such as the anthocyanin, cyanidin glucoside, from Queen Garnet plums [31] and carrageenans from red seaweeds such as *Sarconema filiforme* [32].

This current study has combined partial medial meniscectomy of the right knee [24] together with chronic consumption of fructose with beef tallow for a total of 24 weeks [16] to produce rats with progressive trauma-induced OA and obesity. The results of this study indicate that chronic consumption of beef tallow or its replacement by palmitic or stearic acids led to increased pain, subchondral bone changes and metabolic syndrome parameters compared to replacement of beef tallow with lauric or myristic acids. Similarly, our previous study for 16 weeks in obesity-induced OA reported that lauric acid, the medium-chain SFA, showed preventive effects against diet-induced obesity, cardiovascular and liver complications and OA development, whereas palmitic and stearic acids as long-chain SFA increased these parameters [16]. Since lauric and myristic acids were oxidised faster than palmitic and stearic acids [33], the oxidation rate of different SFA could be inversely related to changes in body fat, and metabolic and organ functions, as well as OA development.

Adipokines may provide the link between biomechanics, behaviour and inflammation as factors in promoting obesity-associated OA, especially since OA is common in non-weight-bearing joints such as hands and shoulders [34]. Beyond mechanical loading, adipokines modulate pro/anti-inflammatory and anabolic/catabolic balance, apoptosis, matrix remodelling and subchondral bone ossification [35,36]. One important source of these cytokines to mediate inflammatory responses is the infrapatellar fat pad [5]. Inflammation then drives sensitisation which is associated with pain severity and may contribute to the transition from acute to chronic pain in knee osteoarthritis. IL-1β is one of the major cytokines involved in the pathogenesis of OA, where it causes inflammatory reactions to the articular cartilage [35,36]. In contrast, IL-10 has chondroprotective effects during OA pathogenesis which induces the synthesis of aggrecan and type II collagen in the cartilage [35,36]. In our study, plasma concentrations of pro-inflammatory cytokines (IL-1β and IFN-γ) were increased and an anti-inflammatory cytokine (IL-10) was decreased in rats fed palmitic or stearic acids, suggesting excessive systemic inflammation. The decreased expression of IL-1β and IFN-γ and increased expression of IL-10 in rats fed lauric or myristic acids suggests a markedly reduced inflammatory response to these SFA. Changes in pro-inflammatory cytokines may also relate to decreased body weight in rats fed lauric and myristic acids; a meta-analysis demonstrated that a 5–10% decrease in body weight improved pain, self-reported disability and quality of life in obese OA patients [37]. Further, other fatty acids, especially omega-3 polyunsaturated fatty acids, also show decreased inflammatory markers and cartilage degradation in animals and humans [38].

One of our major findings was that post-meniscectomy rats, independent of diet group and body weight, had similar Mankin scores. This suggests that body weight did not play a role in the differences in cartilage degradation. The regions of the cartilage were equally damaged in response to the different diets and hence we could not draw any correlation between degeneration of articular cartilage, pain behaviour and inflammatory factors. In contrast, abnormalities in subchondral bone during OA can promote development of joint pain [39,40], with our results showing post-traumatic changes in the bone morphology that were exacerbated by palmitic and stearic acids.

Pain in OA is complex with both neuropathic and nociceptive mechanisms [41]. The behavioural methods to measure stimulus-evoked and non-stimulus-evoked nociception in rodents have been evaluated [26], justifying measurements with different methods as in this study. The behavioural pain responses contributed by activation of spinal microglia and astrocytes show that there may be a positive correlation between distal allodynia and spinal glial cells. The neural damage which arises from the progressive degradation of cartilage induces a chronic state such as neuropathic pain [42]. Neuronal damage caused by the proliferation of microglia in the dorsal regions is one of the main causative agents for the pathogenesis of neuropathic pain [43]. It is reported that spinal glial cells contributed to pain behaviour in monoiodoacetate-induced OA in rats and therapy that alleviated OA pain inhibited activated microglia and reduced astrocyte expression [44,45,46].

In this study, we observed impairments in both pain threshold (PWT and LWT) and grip strength in post-meniscectomy rats fed SFA except HLA and C rats. Pain results from both increased nociceptive inputs from the joints and also central sensitisation of the SC and the brain [44]. Allodynia from central sensitisation could play an important role in the reduction of this pain threshold. The common drivers of nociceptors associated with OA are the subchondral bone, muscle, ligaments and synovium [47,48]. The changes in expression of inflammatory mediators in response to different SFA diets could hold the key to this finding, as HLA rats showed decreased pro-inflammatory cytokines. One major factor in the interpretation of pain behaviour results in the study is lack of predictability of the link between pain and structural changes in the knee clinically, except for the presence of osteophytes [49]. Moreover, clinical studies have shown that weight loss in obese patients with knee OA relieves disability from OA [37]. Nevertheless, more studies are required to elucidate the changes in pain behaviour in response to high-fat diets, in particular related to the content of medium-chain rather than long-chain SFA.

The major limitation of this study is the unknown ability to translate results from this rat model to obese humans with chronic progressive OA pain, and further to non-obese OA patients. The mechanisms of the responses need further investigation as this may provide targeted interventions for pain relief. Possible targets include the local production of inflammatory mediators from the infrapatellar fat pad [5] and changes in the intestinal microbiome [50], both of which may alter OA pain.

## 5. Conclusions

Our results indicate that dietary SFAs differentially influence both development of obesity and pain behaviour in trauma- and obesity-induced OA, with medium-chain SFA having minimal effects on both obesity and pain behaviour in contrast to longer-chain SFA. This suggests that altering dietary SFA to medium-chain SFA may improve both obesity and pain behaviour, probably by altering inflammatory mediators.

## Figures and Tables

**Table 1:** Dietary parameters in rats treated with fatty acids.

Variables	C	H	HLA	HMA	HPA	HSA
Initial body weight, g	340 ± 2^ab^	336 ± 0^b^	340 ± 2^ab^	336 ± 1^b^	344 ± 3^a^	337 ± 1^b^
Final body weight, g	446 ± 10^b^	571 ± 11^a^	460 ± 12^b^	553 ± 15^a^	591 ± 22^a^	492 ± 8^b^
Body weight gain, g	106 ± 10^c^	235 ± 11^a^	120 ± 11^bc^	217 ± 15^a^	247 ± 20^a^	155 ± 8^b^
Food intake, g/d	38.1 ± 1.3^a^	25.7 ± 0.3^cd^	23.8 ± 1.1^d^	27.2 ± 1.1^bcd^	30.7 ± 1.0^b^	29.4 ± 1.5^bc^
Water intake, mL/d	25.6 ± 2.4^b^	23.3 ± 1.3^b^	37.9 ± 2.3^a^	28.8 ± 1.4^b^	34.7 ± 1.0^a^	33.8 ± 0.8^a^
Energy intake, kJ/d	428 ± 14^c^	548 ± 9^b^	571 ± 19^b^	596 ± 23^b^	681 ± 21^a^	654 ± 28^a^
Feed efficiency, g/kJ	0.25 ± 0.02^c^	0.43 ± 0.02^a^	0.21 ± 0.02^c^	0.36 ± 0.02^b^	0.36 ± 0.02^b^	0.24 ± 0.01^c^

All values are mean ± SEM, *n* = 6. Mean values within a row with a different superscript (a, b, c, or d) are significantly different, *p* < 0.05. C, corn starch diet-fed rats; H, high-carbohydrate high-fat diet-fed rats; HLA, high-carbohydrate high-lauric acid diet-fed rats; HMA, high-carbohydrate high-myristic acid diet-fed rats; HPA, high-carbohydrate high-palmitic acid diet-fed rats; HSA, high-carbohydrate high-stearic acid diet-fed rats.

**Table 2:** Metabolic parameters in rats treated with fatty acids.

Variables	C	H	HLA	HMA	HPA	HSA
Abdominal circumference, cm	19.8 ± 0.2^d^	23.6 ± 0.1^ab^	21.1 ± 0.5^c^	22.9 ± 0.4^ab^	23.8 ± 0.6^a^	22.3 ± 0.2^b^
Retroperitoneal fat, mg/mm*	190 ± 25^b^	489 ± 34^a^	201 ± 11^b^	389 ± 42^a^	379 ± 37^a^	272 ± 34^b^
Epididymal fat, mg/mm*	97 ± 8.9^c^	231 ± 15^a^	110 ± 13^c^	221 ± 11^a^	208 ± 17^a^	163 ± 24^b^
Omental fat, mg/mm*	144 ± 18^c^	261 ± 33^a^	133 ± 11^c^	229 ± 19^ab^	207 ± 19^abc^	163 ± 16^bc^
Abdominal fat pads, mg/mm*	431 ± 50^b^	981 ± 76^a^	444 ± 25^b^	840 ± 60^a^	794 ± 60^a^	598 ± 67^b^
Bone mineral density, g/cm^2^	0.185 ± 0.004^ab^	0.193 ± 0.005^a^	0.186 ± 0.003^ab^	0.186 ± 0.004^ab^	0.189 ± 0.003^ab^	0.176 ± 0.004^b^
Bone mineral content, g	12.9 ± 0.5^c^	16.7 ± 0.6^ab^	13.2 ± 0.6^c^	15.3 ± 0.6^b^	17.5 ± 0.70^a^	13.0 ± 0.4^c^
Total fat mass, g	98 ± 13^c^	209 ± 20^a^	105 ± 15^c^	205 ± 20^a^	231 ± 22^a^	129 ± 15^c^
Total lean mass, g	329 ± 5	350 ± 17	348 ± 9	333 ± 19	339 ± 12	359 ± 20
Blood glucose AUC, mmol/L×min	720 ± 39^ab^	773 ± 26^a^	644 ± 24^b^	653 ± 18^b^	720 ± 20^ab^	638 ± 23^b^
Fasting blood glucose, mmol/L	3.5 ± 0.5	4.6 ± 0.2	3.6 ± 0.3	4.0 ± 0.3	4.6 ± 0.3	4.2 ± 0.3
Blood glucose at 120 min, mmol/L	5.2 ± 0.2^ab^	6.3 ± 0.3^a^	4.9 ± 0.3^b^	5.0 ± 0.3^b^	5.3 ± 0.3^ab^	5.2 ± 0.2^ab^
Plasma NEFA, mmol/L	1.46 ± 0.19^b^	3.58 ± 0.56^a^	2.04 ± 0.44^ab^	3.25 ± 0.53^a^	3.47 ± 0.28^a^	3.40 ± 0.56^a^
Plasma triglycerides, mmol/L	0.51 ± 0.04^b^	1.33 ± 0.17^ab^	0.56 ± 0.12^b^	1.71 ± 0.40^a^	1.55 ± 0.32^ab^	1.11 ± 0.23^ab^
Plasma total cholesterol, mmol/L	1.41 ± 0.05	1.61 ± 0.11	1.46 ± 0.05	1.64 ± 0.08	1.63 ± 0.03	1.34 ± 0.08

All values are mean ± SEM, *n* = 6. Mean values within a row with a different superscript (a, b, c or d) are significantly different, *p* < 0.05. C, corn starch diet-fed rats; H, high-carbohydrate high-fat diet-fed rats; HLA, high-carbohydrate high-lauric acid diet-fed rats; HMA, high-carbohydrate high-myristic acid diet-fed rats; HPA, high-carbohydrate high-palmitic acid diet-fed rats; HSA, high-carbohydrate high-stearic acid diet-fed rats; AUC, area under the curve; NEFA, non-esterified fatty acids. *Weight normalised to tibial length.

**Table 3:** Cardiovascular and liver parameters in rats treated with fatty acids.

Variables	C	H	HLA	HMA	HPA	HSA
Systolic blood pressure, mmHg	128 ± 3^b^	153 ± 4^a^	119 ± 4^b^	149 ± 9^a^	152 ± 5^a^	154 ± 4^a^
LV diastolic stiffness constant (ĸ)	20.2 ± 1.2	23.2 ± 1.3	20.6 ± 1.6	23.8 ± 0.5	22.3 ± 1.6	24.2 ± 0.5
LV + Septum wet weight, mg/mm*	21.2 ± 1.0^b^	26.0 ± 1.3^a^	20.9 ± 1.0^b^	25.3 ± 0.9^ab^	23.8 ± 1.4^ab^	23.4 ± 1.1^ab^
RV wet weight, mg/mm*	5.28 ± 0.88	5.31 ± 0.22	5.04 ± 0.43	4.67 ± 0.35	4.78 ± 0.48	5.10 ± 0.24
Liver wet weight, mg/mm*	236 ± 10^b^	345 ± 13^a^	267 ± 17^b^	350 ± 11^a^	325 ± 16^a^	263 ± 3.0^b^
Plasma ALT, U/L	34.1 ± 7.7	28.5 ± 2.1	28.4 ± 7.1	32.6 ± 3.9	21.4 ± 1.5	35.1 ± 6.9
Plasma AST, U/L	78.2 ± 4.8	74.6 ± 8.2	71.9 ± 7.5	80.4 ± 8.7	62.8 ± 1.7	70.5 ± 4.0

All values are mean ± SEM, *n* = 6. Mean values within a row with a different superscript (a or b) are significantly different, *p* < 0.05. C, corn starch diet-fed rats; H, high-carbohydrate high-fat diet-fed rats; HLA, high-carbohydrate high-lauric acid diet-fed rats; HMA, high-carbohydrate high-myristic acid diet-fed rats; HPA, high-carbohydrate high-palmitic acid diet-fed rats; HSA, high-carbohydrate high-stearic acid diet-fed rats; ALT, Alanine transaminase; AST, Aspartate transaminase; LV, left ventricle; RV, right ventricle. *Weight normalised to tibial length.
